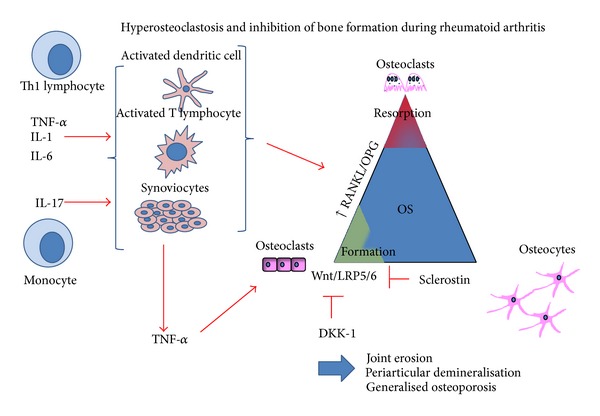# Bone Remodelling Markers in Rheumatoid Arthritis

**DOI:** 10.1155/2014/484280

**Published:** 2014-04-15

**Authors:** Patrice Fardellone, Alice Séjourné, Julien Paccou, Vincent Goëb

**Affiliations:** ^1^Department of Rheumatology, Hôpital Nord, Place Victor Pauchet, 80054 Amiens, France; ^2^INSERM U1088, 1 rue des Louvels, 80000 Amiens, France

## Abstract

Bone loss in rheumatoid arthritis (RA) patients results from chronic inflammation and can lead to osteoporosis and fractures. A few bone remodeling markers have been studied in RA witnessing bone formation (osteocalcin), serum aminoterminal propeptide of type I collagen (PINP), serum carboxyterminal propeptide of type I collagen (ICTP), bone alkaline phosphatase (BAP), osteocalcin (OC), and bone resorption: C-terminal telopeptide of type 1 collagen (I-CTX), N-terminal telopeptide of type 1 collagen (I-NTX), pyridinolines (DPD and PYD), and tartrate-resistant acid phosphatase (TRAP). Bone resorption can be seen either in periarticular bone (demineralization and erosion) or in the total skeleton (osteoporosis). Whatever the location, bone resorption results from activation of osteoclasts when the ratio between osteoprotegerin and receptor activator of nuclear factor kappa-B ligand (OPG/RANKL) is decreased under influence of various proinflammatory cytokines. Bone remodeling markers also allow physicians to evaluate the effect of drugs used in RA like biologic agents, which reduce inflammation and exert a protecting effect on bone. We will discuss in this review changes in bone markers remodeling in patients with RA treated with biologics.

## 1. Inflammation, Joint Erosions, and Bone Mass

Rheumatoid arthritis (RA) is a chronic disease characterized by articular erosions, periarticular bone loss, and chronic inflammation leading to increased risk of osteoporosis [1]. Systemic bone loss associated with RA is multifactorial: glucocorticoids, decrease of physical activity, and the disease itself, particularly when uncontrolled. Bone loss, whether periarticular or systemic, shares, at least partially, similar mechanisms. From the very early stages of RA, bone loss in RA correlates with parameters of inflammation and functional status. Joint erosions measured with Larsen's score are correlated with bone mineral density (BMD) and vertebral deformities [1–5]. Relevant literature on bone remodelling markers in RA patients and the effect of biologic agents on bone remodelling were identified using PubMed database with bone remodelling markers, biologic agents, and rheumatoid arthritis as key words. Systematic reviews and randomized controlled studies were both analyzed.

## 2. Cytokines and Signaling Pathways

Among mechanisms involved in bone loss, proinflammatory cytokines play a major role in explaining hyper-osteoclastosis [6]. The nuclear factor-kappa B (NFkappaB) signaling pathway regulates the expression of hundreds of genes which are involved in diverse processes like inflammation. Receptor activator of NFkappaB Ligand (RANKL) is a membrane protein secreted by osteoblasts that binds to the RANK receptor on osteoclast precursors and provokes maturation of osteoclast cells (Figure 1). Its natural decoy receptor osteoprotegerin (OPG) produced by osteoblasts and stromal cells binds to and confines RANKL and prevents differentiation of osteoclasts [7, 8]. Various proinflammatory cytokines regulate expression of RANKL including tumor necrosis factor (TNF) and interleukin-1 (IL-1) [9–12]. RANKL values can predict the therapeutic response to anti-TNF therapy in RA patients [13], which is not the case for OPG [14], whereas OPG expression is increased in synovium of anti-TNF treated patients: with both infliximab and etanercept. In contrast, RANKL is not influenced by the treatment, showing that the ratio RANKL/OPG is of major importance in regulating bone resorption rather than each of the markers taken alone [15]. Then, it is not surprising that deleterious effects of RANKL on BMD can be prevented by denosumab which is an anti-RANKL monoclonal antibody, increasing BMD and reducing bone turnover in RA patients [16]. Bone formation is also decreased during inflammation as shown in mice. When Dkk-1, a protein that is a member of the dickkopf family, is increased by TNFalpha, it exerts its negative regulation on WNT pathway, blocking osteoblast differentiation and inducing expression of sclerostin (SCL), leading to the death of osteocytes [17]. Higher levels of Dkk-1 are associated with an increased risk of articular erosions independent of age, baseline radiologic features, C-reactive protein (CRP), or disease activity [18]. Interleukin-6 (IL-6) directly induces the production of RANKL by synoviocytes in RA patients through the pathway of janus kinase/STAT, phosphorylation of STAT3 and ERK1/2 [19, 20].

## 3. Bone Remodeling Markers 

Bone matrix is mainly composed of type I collagen and type I collagen telopeptide fragments: I-CTX and ICTP can be measured in both serum and urine. They are very sensitive and specific markers of bone degradation [21, 22]. These two telopeptides are released from type I bone collagen by two different enzymatic systems: (1) ICTP, which is derived from matrix metalloprotease activity (MMP) and is very effective in bone erosions associated with RA, and (2) I-CTX, produced by cathepsin K which on the contrary is involved in systemic bone resorption [23]. In RA the ratio of synovial fluid to serum fluid is increased for ICTP but not for I-CTX. This suggests that ICTP is a sensitive marker of periarticular bone resorption linked to MMPs activity of various cells like synoviocytes [24].

II-CTX is not a bone remodeling marker but a marker of cartilage degradation, even if the two phenomena are closely related in RA. Both bone and cartilage markers are strong and independent predictors of articular erosions. This is illustrated by the COBRA study where high levels of I-CTX and II-CTX measured early in RA predicted an increased risk of further articular damage [25].

## 4. Effect of Biological Agents on Bone Metabolism in RA Patients 

Randomized clinical trials have clearly demonstrated that biological agents are able to prevent partial or even total articular erosions in RA patients. This raises the question of their ability to prevent as well the generalized bone loss associated with inflammation encountered in RA patients. This preventive effect might be demonstrated by variation of bone remodeling markers or bone mineral density (BMD) during the course of treatment.

### 4.1. Anti-TNFalpha

In RA patients, BMD is inversely correlated with serum levels of TNFalpha. Bone formation rather than resorption markers better showed the bone response to anti-TNFalpha [26]. An open cohort study of 102 RA patients treated during one year with infliximab showed both the variations of bone loss at lumbar spine, hip, and hands and the variation of bone remodeling induced by the anti-TNFalpha. When BMD at lumbar spine and hip did not vary, it did incur a significant decrease of 0.8% at the hand (*P* = 0.01), giving evidence that metacarpal cortical bone loss is continuing. In RA treated patients with good EULAR response, variation of BMD was favorable compared to other patients. Serum CTX and RANKL hugely decreased in comparison with baseline values at the same time as the decrease of DAS score and CRP [27].

Another multicentric and prospective cohort study included 48 women with an average age of 54.2 years (±2.1 SD) suffering from severe RA for 10 years (11.4 ± 7.8 SD) who initiated infliximab treatment after the failure of one nonbiologic agent (DMARD). None received bisphosphonates. 77% were under glucocorticosteroid treatment. BMD was not modified during the year of the study but serum I-CTX rapidly and significantly decreased by 30% at the 22nd week before going back to the baseline values. Inversely, PINP values remained stable with a P1NP/CTX ratio in favor of bone formation. The II-CTX, witnessing the cartilage degradation, was not modified in the study group but slightly decreased in patients with values above normal before the biologic agent [21].

In the “BeST” study, four different therapeutic strategies have been evaluated in 218 early RA patients: (1) sequential monotherapy, (2) combined treatment “step up,” (3) combined treatment with glucocorticoids, and (4) treatment with infliximab. BMD was measured at lumbar spine, hip, and hands (from 2nd to 4th metacarpal) after 1 and 2 years. After 2 years for all treated groups there was a bone loss at each of these regions. It should be noted though that there was less bone loss in hands for groups treated with either prednisone or infliximab. Progression of erosions was correlated with the decrease of BMD at both hand and hip regions. The use of bisphosphonates protected only lumbar spine and hip from bone loss [28].

A search in PubMed database to identify studies analyzing the effects of anti-TNFalpha treatments on BMD and bone remodeling markers in RA patients has been able to identify four studies [29–32] in which BMD was either stabilized or increased at lumbar spine (up to 2.8%) or at hip (up to 13.1%). Only one study, concerning 48 patients, was negative [21]. Variations of bone remodeling markers were heterogeneous but showed a slight decrease of resorption and an increase of bone formation.

### 4.2. Anti-iL6 Agents

In vitro, iL-6 blockade reduces osteoclastic differentiation and bone resorption in monocytes cultures stimulated by RANKL or RANKL plus TNFalpha. In transgenic mice, formation of osteoclasts is also strongly inhibited by the anti-inflammatory effects of iL-6 blockade [33].

A pilot study compared 22 healthy nonosteopenic control women with 22 women suffering from active RA treated by perfusions of 8 mg/kg Tocilizumab (TCZ). At baseline, the OPG/RANKL ratio was 5 times lower in RA patients than in controls. Higher levels of Dkk-1, sclerostin, serum betaCTX, and osteocalcin were seen related to a hyper remodeling status and slowing down of bone formation in RA patients. In serum, OPG were negatively correlated with DAS28 score when RANKL levels correlated positively with CRP. After two months, OPG/RANKL ratio was increased when Dkk-1 decreased. Thanks to TCZ, OPG/RANKL increase was particularly significant in 10 patients who were in remission or in a low activity state in contrast with other 12 patients with still active RA. On the other hand, variations of Dkk-1 and sclerostin were similar in both groups. Thus, inflammation suppression by anti-IL-6 rapidly corrects bone homeostasis troubles due to RA [34].

The “OPTION” multicentric randomized pivotal study evaluated the effects of TCZ on bone and cartilage remodeling. They were 416 of 623 patients suffering from moderate suffering from moderate to severe RA who were selected because of an inadequate response to methotrexate. Methotrexate administration alone was compared to the administration of an association of methotrexate and TCZ (4 mg to 8 mg/kg every 4 weeks). TCZ reduced in a dose-dependent way the levels of procollagen type II N-terminal propeptide (PIINP), collagen helical peptide (HELIX-II), and matrix metalloproteinase-3 (MMP-3) after 4, 16, and 24 weeks. Among bone formation markers, only serum aminoterminal propeptide of type I collagen (PINP) significantly increased in comparison with placebo, when 1-CTX and ICTP, markers of bone resorption, decreased [35]. TCZ increases bone formation by increasing the expression of OPG when nonbiological agents have no effect. This is shown in a study of bone biopsies from subjects undergoing a prosthesis replacement of the knee [36]. Finally, TCZ also decreases the levels of dickkopf and normalizes the ratio OPG/RANKL [34]. In RADIATE study TCZ decreased C-reactive protein levels and significantly inhibited cathepsin K-mediated bone resorption, as measured by a decrease in CTX-I with a significant decrease in the CTX-I/OC ratio [37]. Furthermore, the SAMURAI study showed that Tocilizumab monotherapy is more effective at one year in reducing radiological progression in patients presenting with risk factors for rapid progression than in low-risk patients according to four independent predictive markers for progressive joint damage (urinary C-terminal crosslinking telopeptide (uCTX-II), urinary pyridinoline/deoxypyridinoline (uPYD/DPD) ratio, body mass index (BMI), and joint-space narrowing (JSN) score at baseline) [38].

## 5. Rituximab

B lymphocytes enhance bone resorption during RA by secreting RANKL [39]. B lymphocyte depletion obtained by using Rituximab results in a decrease of resorption bone markers [40] and inhibition of RA induced osteoclastosis; this effect is obtained by a reduction of the number of osteoclast precursors in synovium and thus increases the ratio OPG/RANKL in serum [41] and as such could protect BMD. In a prospective study with a follow-up of 3–15 months after Rituximab therapy there was no significant change of the bone formation markers (BAP) and ICTP. However, a nonsignificant tendency of decrease of RANKL (with no change of OPG) and a significant decrease of the bone degradation marker deoxypyridinoline crosslinked collagen I were observed. It appears thus that Rituximab lowered osteoclast activity [42].

## 6. Abatacept 

CTLA4-Ig inhibits linking of CTLA-4 with the monocyte surface receptor CD80/CD86 [43] and could downregulate differentiation and maturation of osteoclasts acting directly on genes [44, 45]. CTLA-4 dose-dependently inhibits RANKL- as well as tumour necrosis factor- (TNF-) mediated osteoclastogenesis in vitro without the presence of T cells [44]. Furthermore, in mice, Abatacept protects against bone loss induced by PTH giving an explanation to the protective effect of Abatacept in RA [46].

## 7. Conclusion 

Bone loss in RA is well documented and is a frequent comorbidity needing diagnosis and prevention. Bone remodeling markers are surrogates to evaluate bone formation, resorption, and further risk of fractures. So far, there is no consensus about their role in helping physicians in a clinical point of view. In addition to specific antiosteoporotic agents, when needed, biologic agents add their own nonspecific effect to protect RA patients against bone loss and osteoporotic fractures by reducing inflammatory-linked bone loss.

## Figures and Tables

**Figure 1:**